# Clinical Presentations of Teenage Outpatients Encountered by General Internists in Japanese Hospitals: A Cross-Sectional Study

**DOI:** 10.7759/cureus.35430

**Published:** 2023-02-24

**Authors:** Keiichiro Kita, Shota Kuroiwa, Mayuko Saito, Maiko Kuroiwa, Azusa Sekijima, Daishi Ogawa, Seiji Yamashiro

**Affiliations:** 1 General Internal Medicine, Toyama University Hospital, Toyama, JPN; 2 Internal Medicine, Kamiichi General Hospital, Kamiichi-machi, JPN; 3 Internal Medicine, Nanto Municipal Hospital, Nanto, JPN

**Keywords:** primary care medicine, adjustment disorder, eating disorders (eds), general medicine, general internist, psychosomatic medicine, stress-related mental disorders, teenage outpatients, adolescents

## Abstract

Introduction

General internists in Japanese hospitals sometimes consult on adolescents. Our university hospital encounters more adolescents with mental health complaints than other city hospitals. Consequently, based on our experience, we hypothesized that psychiatric disorders are significantly more common among teenagers who visit general internists. Therefore, we retrospectively analyzed the clinical profiles of teenage outpatients who visited general internists at three hospitals to test this hypothesis.

Methods

This study included 342 patients aged 13-19 years who visited the Departments of General Internal Medicine at Toyama University Hospital, Nanto Municipal Hospital, and Kamicichi General Hospital between January 2019 and December 2021. Data on age, sex, chief complaint, the period from onset to visit, referral status, and final diagnosis were collected from medical records. We also identified the final diagnoses of 1,375 outpatients from the university hospital during the same period, stratifying them by age. Multiple comparison analyses, Chi-squared tests, and residual analyses were performed to analyze the data.

Results

The number of psychiatric teen patients was significantly higher in the university hospital group than in the other city hospital groups (p<0.01). The incidence of psychiatric disorders, such as stress-related mental disorders, including adjustment and eating disorders (p<0.001), was also significantly higher in the teenage group (13-19 years) than in other age groups. Most psychiatric disorders lead to complaints of physical symptoms.

Conclusions

The potential onset of clinical episodes during consultations with teenage patients can make treating this age group more challenging; thus, they may require care at university hospitals. Furthermore, Japanese general internists in university hospitals encounter patients in their late teens with physical signs more often than in other hospitals. This trend may be unique to general medicine departments (“Sogo-Shinryo”) in Japanese university hospitals. However, if general internists act under primary care principles, they can adequately assist adolescent patients.

## Introduction

Japan’s public health insurance system ensures health services to most of the population. Although access regulations are gradually progressing, patients are essentially free to visit all types of care providers, from solo practitioners to hospital specialists. Therefore, general internists in Japanese hospitals encounter patients from adolescence (usually over fifteen) to adulthood with miscellaneous complaints [1]. Adolescent patients undergo rapid physical and psychosocial developmental changes, resulting in exceptional communication and management difficulties [2, 3]. Furthermore, psychological problems can cause somatic symptoms, making it more challenging to provide adequate care. Based on our experience, we hypothesized that teenage patients presenting to the general medicine department of our university hospital have a higher prevalence of psychiatric disorders. However, it was unclear if this experience was unique to our institution. Therefore, we retrospectively analyzed the clinical profiles of teenage outpatients who visited general internists at three hospitals in the same region to clarify this question and investigate mental health disorders in adolescents.

This article was previously posted to the medRxiv preprint server on the Research Square preprint server on June 1, 2022.

## Materials and methods

Study design and participants

This observational study was jointly conducted by one university hospital and two city hospitals based in the Toyama Prefecture, Japan: Toyama University Hospital, Nanto Municipal Hospital, and Kamiichi General Hospital. These hospitals are among the few core hospitals in the same region with an outpatient general medicine department staffed by board-certified general internists. Toyama University Hospital (612 hospital beds) is a Japanese National University Hospital that mainly provides advanced medical care, but the Department of General Internal Medicine offers primary care. Nanto Municipal Hospital (175 hospital beds), located in the west of Toyama, and Kamiichi General Hospital (199 beds), located on the east side, are public city hospitals that provide community health care. General internists staff the outpatient departments of all three hospitals. Four physicians at Toyama University Hospital, four at Nanto Municipal Hospital, and six at Kamiichi General Hospital are certified in their specialty by The Japan Primary Care Association. Overall, 615 new walk-in outpatients aged 13−19 years who presented to the general medicine outpatient departments at the three hospitals between January 2019 and December 2021 were included in this study. However, we excluded 205 teenage patients for inadequate medical record entries and 68 asymptomatic teenagers who visited for follow-up medical checkups for usually mild anomalies detected during a routine physical examination. Finally, 342 teenage patients from the three hospitals were included in the analyses. Their data were retrospectively collected from each hospital’s medical charts, including age, sex, referral status, chief complaint, the period from onset to visit, and final diagnosis. In addition, we identified 2,160 new outpatients over 20 years of age from the university hospital who presented during the same period to compare the distribution of diseases among ages. We excluded 632 cases with limited information, 87 for re-examining health checks, and 66 because of triage for suspected COVID-19. Finally, we enrolled 1,375 patients over 20 years of age, collecting their final diagnoses from their medical records.

Classification of symptoms and final diagnoses

We used the term “digestive symptoms” for abdominal pain with various GI symptoms, such as nausea and diarrhea; “respiratory symptoms” for cough, sputum, and dyspnea; and “cardiovascular symptoms” for palpitations and chest pain. The patients were grouped into organic, functional, and psychiatric groups based on the final diagnosis, defined as follows: 1) organic diseases: detectable and observable organ changes, such as infectious diseases; 2) functional diseases: abnormal changes in organ function but not structural changes, such as migraines; and 3) psychiatric disorders: mental problems clinically diagnosed by a psychiatrist and/or general internist using the diagnostic criteria of the Diagnostic and Statistical Manual of Mental Disorders, Fifth Edition [4]. We adequately examined the psychiatric patients to rule out organic diseases. In addition, we used the patients’ medical histories to roughly categorize the patients into two groups based on the period from the onset of symptoms to ambulatory visits: ≤1 month and >1 month.

Statistical analyses

Since this was an observational study without intervention, the sample size was judged sufficient to allow for group-by-group comparisons, and power analyses were not conducted. Multiple comparison analyses using Tukey’s Honest Significant Difference, Chi-squared, residual analysis, and Fisher’s exact tests were performed using Bell Curve for Excel (version 3.20). Statistical significance was set at p<0.05.

Ethics approval

The ethical review board of Toyama Hospital approved this study (approval number: R2021123). The participants had indirect opt-out opportunities through announcements on the three hospital bulletin boards, which were deemed an acceptable method of obtaining consent. Furthermore, as an ethical consideration, all patient data were anonymized during collection from the medical records, precluding analysts from matching registration numbers with individuals.

## Results

Table 1 shows the characteristics of enrolled adolescent patients. This study included 148 male and 194 female patients aged 13-19 years (male/female ratio: 1:1.31). The mean age was 16.6±1.56 years for male patients and 16.7±1.48 years for female patients. Most patients under 14 years of age were referred to the general internists of the hospitals by pediatricians. The university hospital group had fewer male patients (p=0.0156) and a significantly higher referral rate (p<0.01) than the other hospitals. Most patients in the two city hospitals visited the outpatient departments within one month of symptom onset, but over half of the cases at the university hospital visited the department more than one month after symptom onset. Almost all patients visited the hospitals with somatic symptoms, and some had more than one symptom. Digestive symptoms, fever, and headache were the most prevalent chief complaints in all three hospitals. Respiratory symptoms were also common in both city hospital groups but were minor in the university hospital group (p<0.01). Additionally, arthralgia/myalgia was more common in the Nanto Municipal Hospital than in the other hospitals (p<0.01).

**Table 1:** Background characteristics of the enrolled patients. *, **Multiple comparison analysis by Tukey’s Honest Significant Difference. *: p=0.0156, **: p<0.01. ***Residual analysis by Chi-squared test examining the difference between the actual and expected value at each cell. ***+: greater than the expected value, p<0.01; ***-: less than expected value, p<0.01.

	Toyama University Hospital (N=84)		Nanto Municipal Hospital (N=97)		Kamiichi General Hospital (N=161)	
sex, female （n, %)	59*	70.20%	50	51.50%	85	52.80%
Age (y), mean±SD	16.9±1.5		16.2±1.6**		16.9±1.4	
Age group (y), (n) [%]						
13	1	1.20%	7	7.20%	0	0.00%
14	4	4.80%	10	10.30%	10	6.20%
15	9	10.70%	10	10.30%	15	9.30%
16	21	25.00%	31	32.00%	36	22.40%
17	15	17.90%	20	20.60%	45	28.00%
18	19	22.60%	10	10.30%	33	20.50%
19	15	17.90%	9	9.30%	22	13.70%
Referral (n, %)	52** (61.9)		7 (7.2)		12 (7.5)	
Visit <1 month from onset (n, %)	37* (44.0)		76 (78.4)		149** (92.5)	
Chief Complaints （n, %)						
Digestive symptoms	24	28.60%	26	26.80%	65	40.40%
Fever	13	15.50%	22	22.70%	66	41.00%
Headache	11	13.10%	13	13.40%	39	24.20%
Fatigue	8	9.50%	8	8.20%	18	11.20%
Sleep disturbance	8	9.50%	1	1.00%	1	0.60%
Dizziness	7	8.30%	2	2.10%	9	5.60%
Cardiovascular symptoms	7	8.30%	1	1.00%	7	4.30%
Unable to attend school	7	8.30%	2	2.10%	1	0.60%
Respiratory symptoms	5***-	6.00%	31	32.00%	42	26.10%
Arthralgia/myalgia	3	3.60%	14***+	14.40%	8	5.00%
Urinary symptoms	3	3.60%	5	5.20%	8	5.00%
Tremor	3	3.60%	0	0.00%	2	1.20%
Dermatologic problems	2	2.40%	6	6.20%	8	5.00%
traumatic accident	0	0.00%	12	12.40%	0	0.00%
Others	19	22.60%	8	8.20%	6	3.70%

Table 2 presents the final diagnosis for each patient group. The proportion of patients with organic diseases was higher at the two city hospitals than at the university hospital. Acute respiratory infections, such as acute bronchitis and influenza, were prevalent in the two city hospital groups. Furthermore, generalists in the Nanto Municipal Hospital treated more outpatients with mild traumatic injuries than the other hospitals because of the lack of orthopedic surgeons. Alternatively, patients in the university hospital group had some severe conditions, such as acute leukemia, eosinophilic gastroenteritis, and spontaneous intracranial hypotension. Adjustment disorders were the most common psychiatric diseases, followed by anorexia nervosa and anxiety disorders. Nine of the ten patients complaining of being unable to attend school were female; six were diagnosed with psychiatric disorders, and three were diagnosed with functional diseases. Eleven female psychiatric patients of the university hospital mentioned academic stress, including an academic performance at school and preparation for entrance examinations. However, none had socioeconomic problems, and they attended the best college-prep schools in their area.

**Table 2:** Final diagnosis of each patient group. OD/NMD: Orthostatic dysregulation/Neurally mediated syncope; FD: Functional dyspepsia. *Residual analysis based on the Chi-squared test examining the difference between the actual and expected value at each cell. *+: greater than the expected value, p<0.01; *-: less than the expected value, p<0.01.

	Toyama University Hospital (N=84)				Nanto Municipal Hospital (N=97)				Kamiichi General Hospital (N=161)			
Diagnosis	n	(%)	male	female	n	(%)	male	female	n	(%)	male	female
Somatic diseases	58				92				158			
Organic diseases	39*^-^	46.40%			77	79.40%			134*^+^	83.20%		
Infectious enterocolitis	6	7.10%	4	2	4	4.10%	1	3	25*^+^	15.50%	12	13
Iron deficiency anemia	5	6.00%	0	5	0	0.00%	0	0	1	0.60%	0	1
Upper respiratory infections	4*	4.80%	2	2	24	24.70%	12	12	53*^+^	32.90%	27	26
Acute cystitis/pyelonephritis	3	3.60%	0	3	6	6.20%	0	6	4	2.50%	1	3
Infectious monoarthritis	3	3.60%	3	0	2	2.10%	1	1	1	0.60%	1	0
Dermatologic problems	2	2.40%	1	1	6	6.20%	4	2	8	5.00%	5	3
Asthma	0	0.00%	0	0	3	3.10%	2	1	4	2.50%	2	2
Thyroid diseases	1	1.20%	0	1	4	4.10%	0	4	0	0.00%	0	0
Mild traumatic injury	0	0.00%	0	0	13	13.40%	10	3	0	0.00%	0	0
Others	15	17.90%	3	12	15	15.50%	7	8	38	23.60%	20	18
Functional diseases	19	22.60%			15	15.50%			24	14.90%		
OD/NMD	8	9.50%	4	4	1	1.00%	0	1	7	4.30%	4	3
Migraine	3	3.60%	1	2	0	0.00%	0	0	4	2.50%	1	3
Irritable bowel syndrome/FD	3	3.60%	0	3	9	9.30%	7	2	7	4.30%	1	6
Tension type headache	0	0.00%	0	0	2	2.10%	1	1	2	1.20%	1	1
others	5	6.00%	3	2	3	3.10%	1	2	4	2.50%	2	2
Psychiatric diseases	26*^+^	31.00%			5	5.20%			3*^-^	1.90%		
Adjustment disorder	11	13.10%	2	9	1	1.00%	1	0	1	0.60%	0	1
Anorexia nervosa	5	6.00%	0	5	2	2.10%	0	2	0	0.00%	0	0
Anxiety disorder	3	3.60%	1	2	1	1.00%	0	1	0	0.00%	0	0
Somatic symptom disorder	2	2.40%	0	2	0	0.00%	0	0	0	0.00%	0	0
Obsessive-Compulsive disorder	1	1.20%	0	1	0	0.00%	0	0	0	0.00%	0	0
Mood disorder	1	1.20%	0	1	0	0.00%	0	0	0	0.00%	0	0
Others	3	3.60%	1	2	1	1.00%	0	1	2	1.20%	0	2

Moreover, the university hospital received more psychiatric teen patients than the two city hospitals (p<0.01). At the university hospital, 16 patients currently or previously attended a psychiatric clinic, and eight were newly referred to psychiatrists by generalists. Also, the adolescent age group (13-19 years) had a significantly higher prevalence of psychiatric disorders than the other age groups who visited the general medicine department at the university hospital during the same period (p<0.001, Table 3).

**Table 3:** Disease distributions by age of the university hospital patients. *Residual analysis based on the Chi-squared test examining the difference between the actual and expected values at each cell.

Age range	Psychiatric diseases		Non-psychiatric diseases		Total		P-value*
	Number	(%)	Number	(%)	Number	(%)	
13-19	26	-31	58	-69	84	-100	<0.001
20s	10	-5.8	161	-94.2	171	-100	0.055
30s	27	-16.4	138	-83.6	165	-100	0.004
40s	25	-11.8	186	-88.2	211	-100	0.329
50s	17	-8.8	176	-91.2	193	-100	0.558
60s	17	-10	153	-90	170	-100	0.802
70s	16	-5.4	281	-94.6	297	-100	0.01
80s~	5	-3	163	-97	168	-100	0.001

Additionally, we classified the university hospital patients into psychiatric and non-psychiatric (organic and functional diseases) subgroups and compared their various characteristics (Table 4). The psychiatric disease group took a significantly longer time (over one month) from onset to visit the hospital than the non-psychiatric group (p=0.009). The psychiatric disease group also had more female patients than the non-psychiatric group. However, the difference was statistically insignificant, as were age and referral status differences between the two groups. Notably, the psychiatric diseases group complained that they could not attend school because of inadequate physical conditions; they tended to have more symptoms than the non-psychiatric group, such as digestive symptoms, fatigue, palpitations, and fever, but the difference was statistically insignificant.

**Table 4:** Characteristics of the university hospital patients with and without psychiatric diseases Comparisons were performed by Chi-squared or Fisher’s exact tests. *: p<0.05

	Psychiatric disease		
	Present (26)	Absent (58)	P-value
Age	16.9±1.07	16.9±1.65	0.817
Female	22 (84.6%)	37 (63.4%)	0.054
Referral	14 (53.8%)	38 (65.5%)	0.308
>1 month from onset to the hospital visit	21 (80.8%)	26 (44.8%)	0.009*
Chief complaints			
Digestive symptoms	9 (34.6%)	15 (25.9%)	0.412
Unable to attend school	5 (19.2%)	2 (3.4%)	0.027*
Cardiovascular symptoms	4 (15.4%)	3 (5.2%)	0.195
Fatigue	3 (11.5%)	5 (8.6%)	0.698
Dizziness	2 (7.7%)	5 (8.6%)	1
Headache	1 (3.8%)	10 (17.2%)	0.16
Fever	1 (3.8%)	12 (20.7)	0.056

## Discussion

The university hospital patient group had significantly more psychiatric teen patients than the two city hospitals. Furthermore, at the university hospital, the adolescent age group (13-19 years) had a considerably higher prevalence of psychiatric disorders than the other age groups. The primary psychiatric disorders were stress-related mental disorders, such as adjustment and eating disorders. General internal medicine departments in Japanese university hospitals usually receive many referrals from clinics and other hospitals [1]. Therefore, they often encounter challenging cases that do not fit with the biomedical models [5-8]. Nevertheless, it is unique that a higher rate of psychiatric diseases was observed in teenage patients than in any other age group at the same institution. Generally, adolescence, ranging from ages 10 to 19 years, is a period characterized by dynamic change across multiple systems [3] and vulnerability and sensitivity to negative and positive experiences [9, 10]. Furthermore, adolescence is critical from a mental health perspective because many major mental disorders develop during this period [11]. For example, eating disorders usually begin in the 10-20 year age range [12], and anxiety disorders start in childhood, adolescence, or early adulthood, peaking in middle age [13]. However, adjustment disorders are found in all cultures and all age groups and are not specific to teenagers [14]. In addition to the biological and psychological characteristics of adolescence mentioned above, social stresses of school life and low self-esteem may affect psychosomatic disorders. School life, a period of group life with those of the same age, can be a stressful environment for some students. Literature from developed countries reports that school-related stress affects students’ physical and mental health [15, 16]. Additionally, university-preparatory schools are competitive environments in Japan. Therefore, low self-esteem positively correlates with physical and mental problems among teenagers and is a notable risk factor for developing eating disorders and other psychological issues [17-19].

Our data also showed that most patients with psychiatric disorders in the university hospital group were female, similar to many studies that have reported more stress-related mental illnesses in women. For example, studies in western countries indicated that subjective health complaints and psychosomatic symptoms are more prevalent in adolescent female patients [20, 21]. Other studies have shown that adolescent females tend to have lower self-esteem and more negative assessments of their physical characteristics and intellectual abilities than males [22]. We did not objectively evaluate the patient’s self-esteem in this analysis; however, the assessment of the patients derived from these previous studies is consistent with our experience. These findings may partially explain why psychosomatic disorders occur in female students at high-level college preparatory schools. Improving self-esteem and developing a sense of coherence may be essential for these patients [23-25]. This analysis found digestive symptoms common in the psychiatric and somatic disease groups. One study on physical symptoms in psychiatric patients visiting the general medicine department of a Japanese university hospital reported that palpitation, dyspnea, tiredness, and headache were more likely in psychiatric disorders [8]. Contrarily, several reports in the literature, specifically regarding teenage patients, reported that abdominal pain is as common as fatigue and headache [26-29]. In the case of teenage patients with these symptoms, a long interval from the onset may be a clue indicating psychosomatic illness. In addition to the general characteristics of teenagers and their living situation, these clinical presentations may make this patient population more challenging to manage; consequently, healthcare providers may direct them toward the area’s university hospital. When managing stress-related mental illnesses such as adjustment, eating, and anxiety disorders, environmental coordination and psychological support are more critical than pharmacotherapy. Therefore, general internists who encounter teenagers should develop their interpersonal communication skills and awareness of developmental processes with this generation, which is crucial in the early detection of problems, emotional support, and effective disease management [30]. Our study is the first to investigate the clinical characteristics of teenage outpatients encountered by general internists working in Japanese hospitals. However, it has several limitations. First, this is a small-sized retrospective observational study conducted in only part of Japan. Therefore, results from this number of cases may vary across regions and hospitals. Second, we did not confirm the outcomes of the excluded patients. Therefore, further, more extensive studies are needed to clarify these issues.

## Conclusions

In Japan, older teenagers (i.e., those over 15 years) visit a general internist, not a pediatrician. Our study indicated that many of their problems are transient mild somatic cases. Our results also suggest that Japanese general internists in university hospitals encounter patients in their late teens with physical signs more often than other hospital types, which may be a unique feature of general medicine departments (“Sogo-Shinryo”) in Japanese university hospitals. Since stress-related diseases are often difficult to distinguish from organic conditions owing to similarities in their clinical presentations, objective and careful monitoring of the patient’s clinical course should be performed rather than rushing judgment. Therefore, building a solid patient-physician relationship based on communication that considers teenagers’ psychosocial characteristics and basic physical examinations that do not rely on excessive laboratory and imaging testing is essential. In doing so, general internists acting under the principles of primary care (i.e., comprehensiveness, continuity, accessibility, comprehensiveness, coordination, continuity, and accountability) can play a considerable role in the care of adolescent patients.
